# Primary omental ectopic pregnancy: A rare case report

**DOI:** 10.1002/ccr3.8063

**Published:** 2023-10-17

**Authors:** Bishal Budha, Kritika Jha, Dhiraj Poudel, Saroj Pokharel, Suraj Aryal, Satish Bajracharya, Bishweshwar Joshi, Asmita Ghimire

**Affiliations:** ^1^ Maharajgunj Medical Campus, Institute of Medicine Tribhuvan University Maharajgunj Nepal; ^2^ Department of Obstetrics and Gynaecology, Maharajgunj Medical Campus Tribhuvan University Maharajgunj Nepal

**Keywords:** ectopic pregnancy, laparotomy, Nepal, omental

## Abstract

Primary abdominal pregnancy is the rarest form of ectopic pregnancy in which the fertilized ovum is directly implanted into peritoneal cavity. This condition poses significantly high risk of maternal morbidity and mortality. Here, we present a case of 5 weeks of primary omental pregnancy managed by laparotomy.

## Graphical abstract

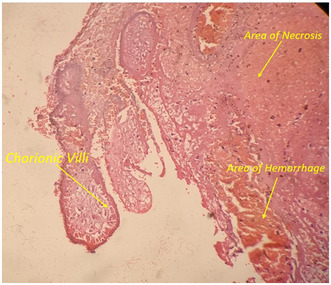

## 1 INTRODUCTION

Abdominal ectopic pregnancy is an extremely rare condition afflicting 1 in 10,000 births, but has significantly high mortality rate up to 20% (7.7 times more than tubal pregnancy and 89.8 times higher than intrauterine pregnancy).^1^ The high risk of maternal morbidity and mortality is due to increased risk of torrential intraperitoneal hemorrhage following partial or complete detachment of placenta from abdominal cavity.^2^ Accurate preoperative localization helps to minimize blood loss while doing surgery. The condition contributes nearly 1% of total ectopic pregnancies and is classified into primary and secondary types.^3^ Primary pregnancy is so rare that its existence is questionable. It occurs after direct implantation of fertilized ovum into peritoneum or any organs of abdominal cavity, whereas secondary pregnancy occurs when the conceptus escapes through ruptured tubes, ovaries, or uterus.^4^ The diagnosis of primary ectopic pregnancy necessitates fulfilling Studdiford's criteria.^5^ We report the case of early abdominal pregnancy at 5 weeks.

## 2 CASE PRESENTATION

A 21‐year‐old unmarried woman presented to the emergency department with a complaint of dull aching, non‐radiating lower abdominal pain and per vaginal bleeding for 1 day. It was associated with five episodes of non‐projectile vomiting with a background history of amenorrhea for the past 4 weeks. There was no history of UPT done either at home or at health center. She was a non‐smoker and had no history of previous gynecological or abdominal surgery, pelvic inflammatory disease, or history of insertion of intrauterine devices.

On presentation to the emergency department, blood pressure was 100/60 mm Hg, heart rate was 108 beats per minute, and respiratory rate was 20 breaths per minute. On examination, she had mild pallor, and on abdominal examination, abdomen was mildly distended and tenderness was noted on lower abdomen. On per speculum examination of genitalia, cervix was smeared with blood and bulky uterus was appreciated on bimanual examination.

**FIGURE 1:**
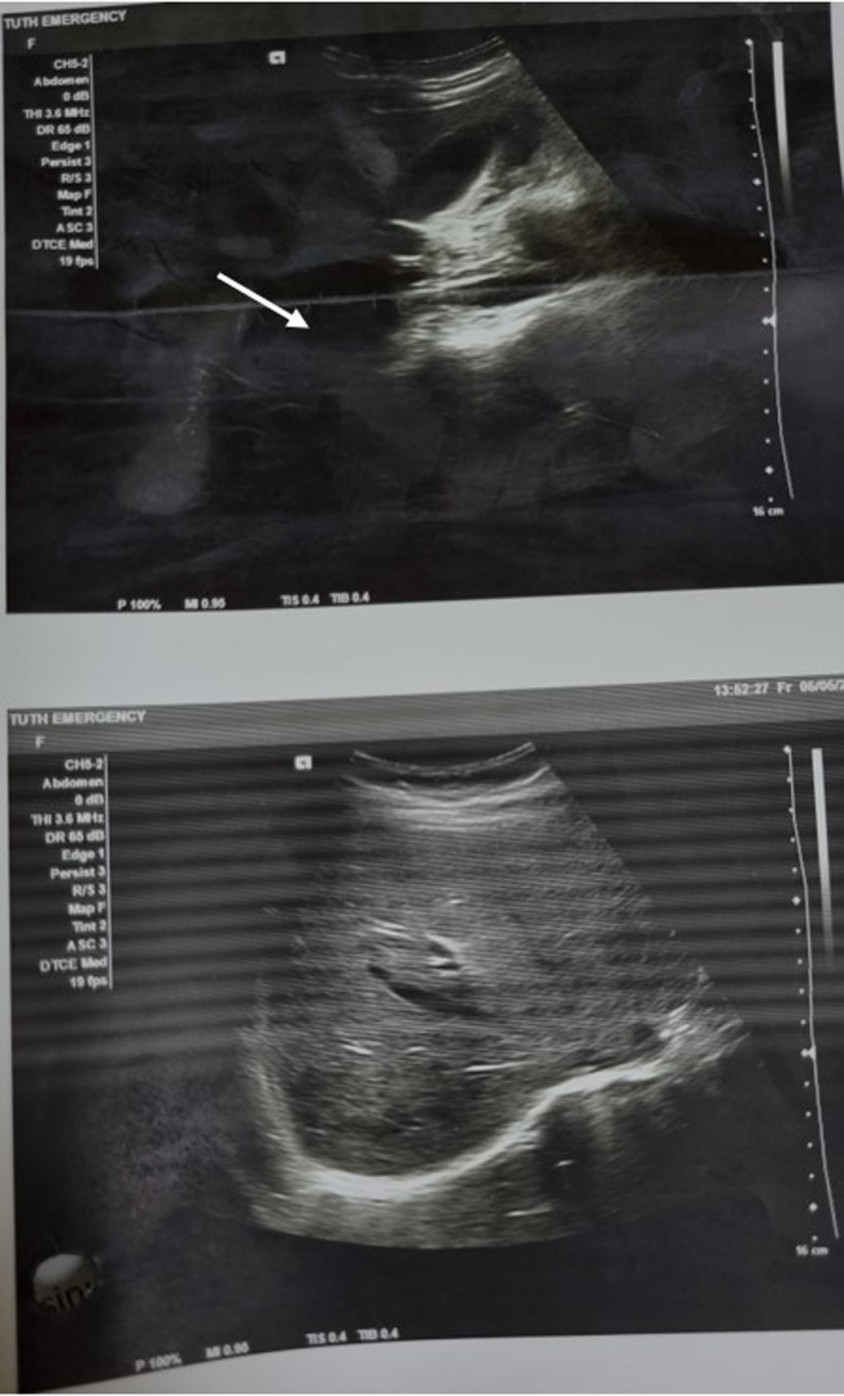
USG abdomen showing moderate fluid collection in the peritoneal cavity (white arrow).

**FIGURE 2:**
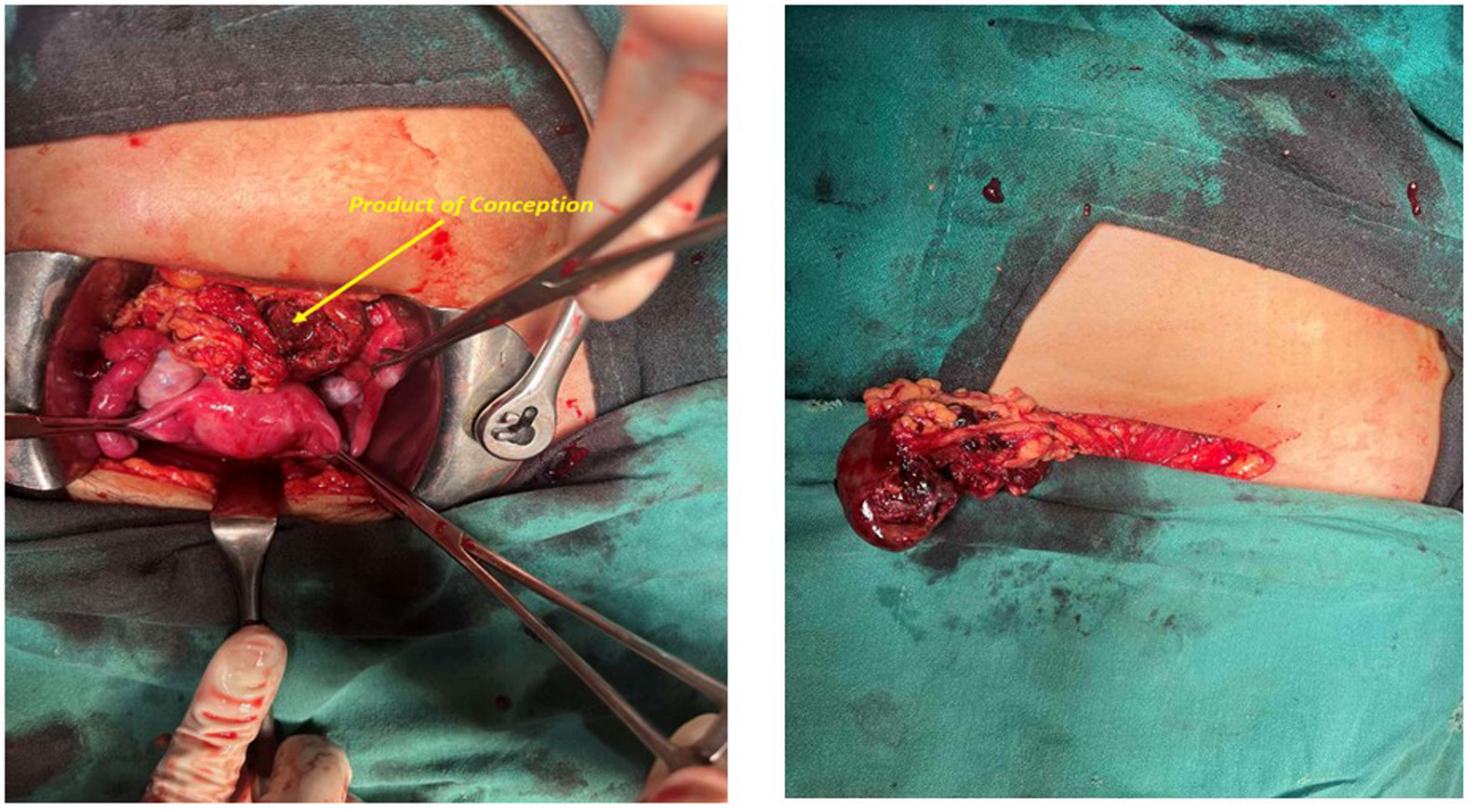
Laparotomy showing product of conception with approximate dimension of 6 cm × 4 cm.

**FIGURE 3:**
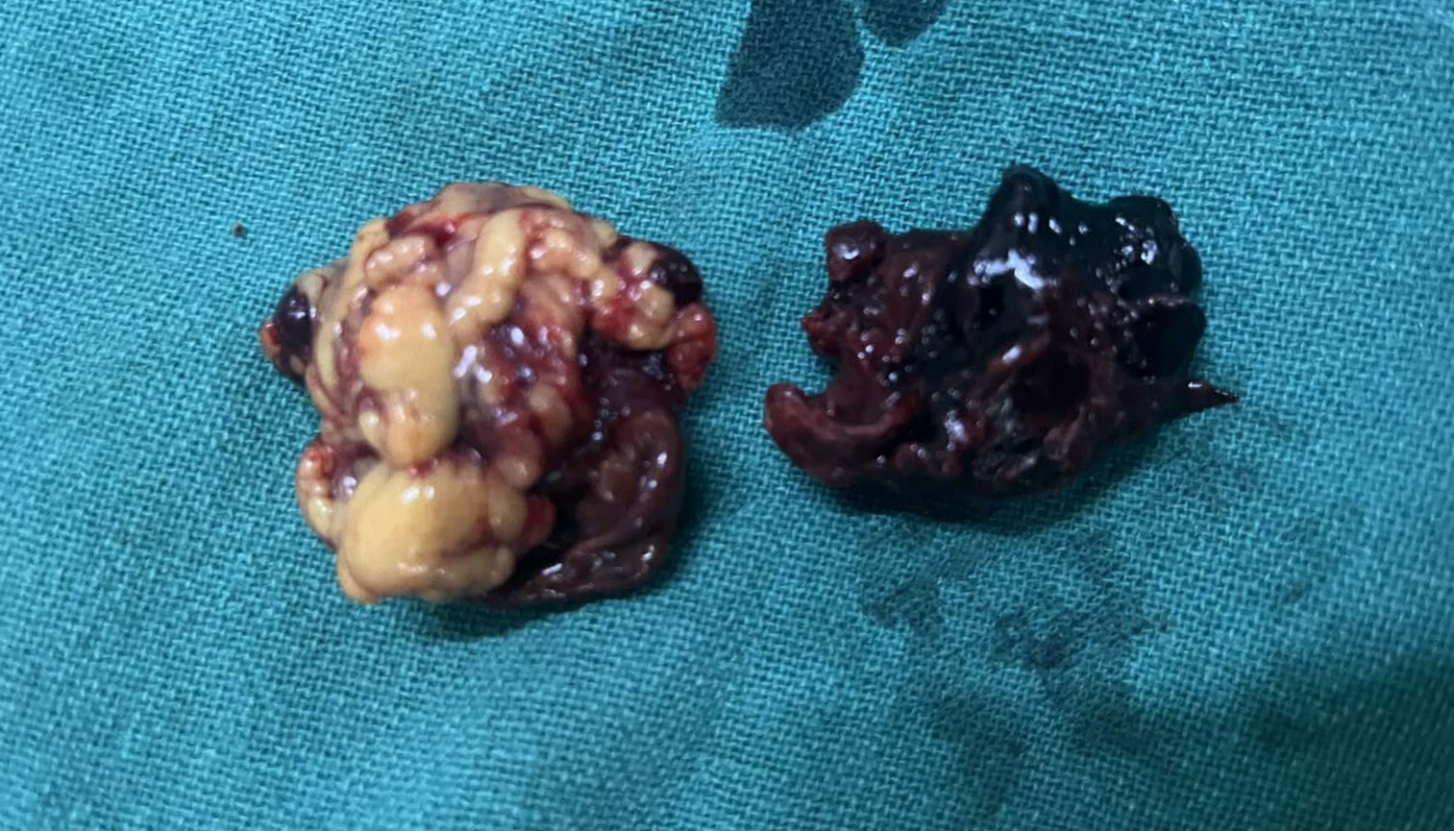
Intra‐abdominal clots with attached omentum.

**FIGURE 4:**
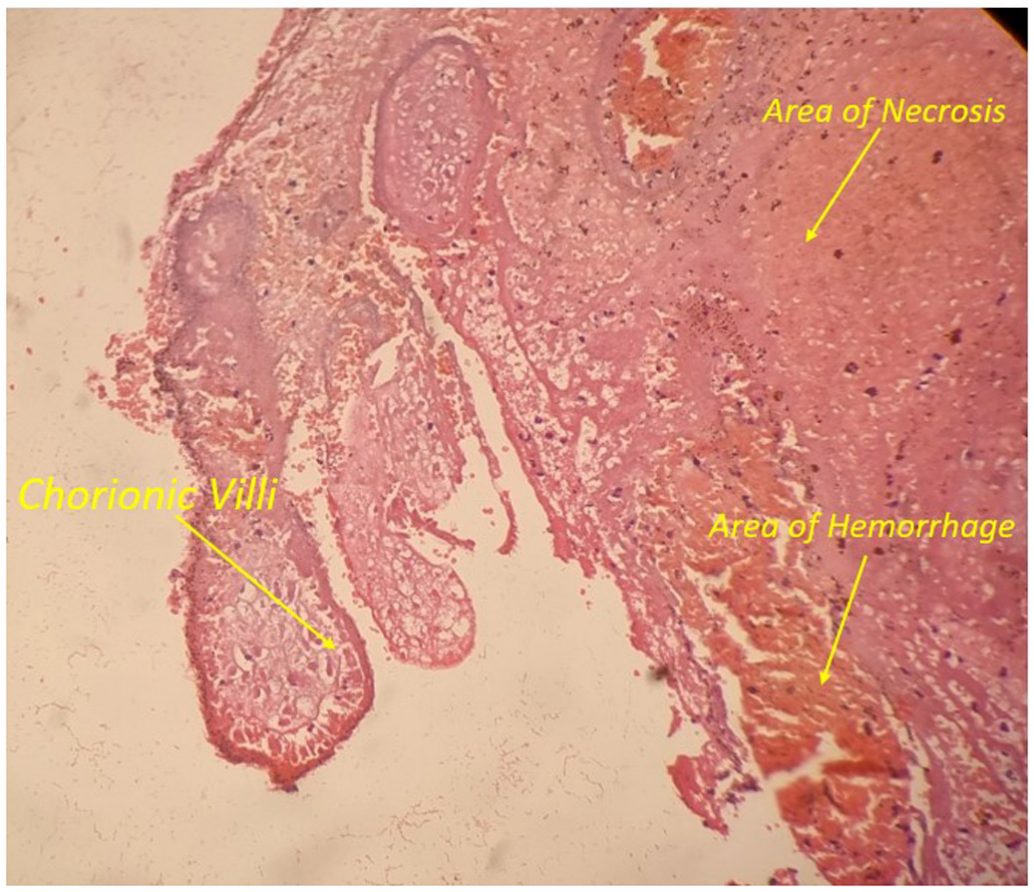
Histologic examination (hematoxylin and eosin staining) showing chorionic villi lined by trophoblastic cells with extensive area of hemorrhage and necrosis.

In the emergency department, crystalloids were started through intravenous line. Blood was drawn, urine sample was collected, and investigations were sent which were reported as urine pregnancy test as “positive,” hemoglobin: 8.6 gm%, total leukocyte count: 12,800 per mm^3^ with polymorphonuclear cells of 84%. Blood grouping was done and reported as O positive following which blood bank was contacted, two pints of packed red cells were arranged, and cross matching was done. Emergency ultrasonography of abdomen and pelvis showed moderate free fluid in the abdomen which on needle aspiration revealed hemorrhagic fluid (Figure 1). The patient was shifted to the operation theater and emergency laparotomy was performed which confirmed the hemo‐peritoneum with content of approximately 500 mL of free fluid and approximately 200 g of blood clots (Figures 2 and 3). Product of conception like material with dimension of 6 cm × 4 cm was found attached to the greater omentum. On inspection intra‐abdominally, the uterus, bilateral fallopian tubes, and bilateral ovaries were found to be intact. The free fluid was drained out by suctioning, clots were removed, and the POC like material was resected completely, to be sent later for histopathological examination. Histopathological examination of the collected tissue revealed extensive areas of hemorrhage, necrosis, neutrophilic infiltration, and sclerosed villi (Figure 4). It was negative for hydatidiform mole and malignancy. The diagnosis of abdominal pregnancy in the omentum was thus confirmed.

## 3 CASE DISCUSSION

Ectopic pregnancy is a condition where the ovum fertilized by spermatozoa gets implanted and develops outside of the endometrial cavity. Sites of common implantations are uterine tubes, ovaries, and intra‐abdominal cavity. Its incidence is nearly 1% of all pregnancies in developed countries and 1 to 3% in African and Asian developing countries.^6^ Approximately 95% of ectopic pregnancies occur in fallopian tube (70% in ampulla, 12% in isthmus, 11% in fimbriae, and 2%–3% in the interstitium), 3% in ovaries and 1% in the abdomen.^7^ Abdominal pregnancy albeit rare is a life‐threatening issue where implantation of fertilized ovum occurs in the peritoneum or peritoneal organs. Abdominal pregnancy is further classified as primary and secondary. Secondary abdominal pregnancy occurs after defect in the passage—rupture of uterus or tubes or the ovaries—of fertilized egg through tubes or ovaries leading to implantation in abdomen, whereas primary abdominal pregnancy is exceptional, occurs by direct implantation.^8^ Diagnosis is made by taking Studdiford's criteria into consideration. Studdiford's criteria were established in 1942 for the diagnosis of abdominal pregnancy. The presence of intact fallopian tubes and ovaries, absence of any utero‐peritoneal fistulae, and pregnancy early enough in gestation to eliminate the possibility of secondary implantation after primary nidation of the tube prove the diagnosis of primary abdominal pregnancy.^3^, ^5^

Various risk factors are established throughout the years in the background of ectopic pregnancy. Prior tubal surgery, prior ectopic pregnancy, pelvic inflammatory disease, sexually transmitted disease, current smoking, in utero diethylstilbestrol exposure, low socioeconomic status, recent use of progesterone‐only pills, and intrauterine devices, myomata, and history of allergy all increases the risk of ectopic pregnancy.^9^, ^10^ Ectopic pregnancy is more common in developing countries because of high prevalence of these risk factors. However in our case, none of the aforementioned risk factors were identified.

The clinical feature of abdominal pregnancy is highly variable and can differ from that of tubal pregnancy. These include abdominal pain, scanty per vaginal bleed in the back ground of amenorrhea. Severe abdominal pain is one of the consistent findings in the case of abdominal pregnancy.^10^, ^11^ In this case, our patient presented with severe lower abdominal pain with PV bleeding. Although ultrasound examination is the preferred diagnostic method, only 50% of early abdominal pregnancy cases can be detected through this procedure. In this particular case, abdominal ultrasonography revealed moderate free fluid in the abdomen, which was confirmed to be hemorrhagic fluid upon aspiration. After laparotomy, intact fallopian tubes and ovaries were found with no utero‐peritoneal fistulae. Since the gestational age of less than 20 weeks of gestation is considered early, we could rule out the possibility of secondary implantation as the gestational age in this case was only 5 weeks. Furthermore, for the evaluation of ectopic pregnancy, non‐contrast MRI using T2‐weighted imaging is considered to be sensitive, specific, and accurate.^12^ However, due to the poor hospital setting and deranged vitals of patient at the time of the presentation, MRI could not be done.

The management of abdominal pregnancy depends on the estimated gestational age at presentation and clinical presentation. There are three modalities of treatment in case of ectopic pregnancy, that is, expectant management, medical management, and surgical intervention. Expectant management is used mainly in tubal ectopic pregnancy.^13^ Medical management is recommended specifically for abdominal pregnancies located in the liver or spleen, as surgical intervention in such cases can lead to significant bleeding.^14^ Most common treatment modality of choice for abdominal pregnancy is surgery where laparotomy and laparoscopic surgery are available options. Laparotomy was considered safer than laparoscopic procedure due to the risk of uncontrollable perioperative hemorrhage from the implantation site but in cases where the implantation site allows non‐surgical excision and in cases of early diagnosis of abdominal pregnancy (less than 12 weeks), laparoscopic surgery is more preferred.^15^, ^16^ Even though our case was diagnosed earlier than 12 weeks of gestation, laparotomy was preferred to laparascopy because the site of pregnancy was not known precisely before the procedure. Thus, laparotomy was used as diagnostic and a therapeutic procedure in our case. Many cases can have negative laparotomy but in our case, we could identify the gestational sac intraoperatively and managed it properly.

Risk of torrential perioperative bleeding always persists. Thus, complete removal of the placenta should be done only after identification of blood supply and its ligation.^17^ Since our patient was at 5 weeks of gestation (early abdominal pregnancy), the risk of bleeding was significantly reduced.

The establishment of early diagnosis of ectopic pregnancy in poor resource setting in rural area of underdeveloped countries like Nepal is a big challenge. It can be improved by the education of healthcare workers and early referral system with low threshold after recognition of risk factors and awareness of atypical presentation.

## 4 CONCLUSION

Preoperative diagnosis of abdominal pregnancy is low. Therefore, abdominal pregnancy should be considered as a differential diagnosis while performing laparotomy for hemo‐peritoneum with suspicion of ruptured ectopic pregnancy. Laparoscopy and laparotomy are the surgical options for diagnostic and therapeutic purposes.

## AUTHOR CONTRIBUTIONS

**Bishal Budha:** Conceptualization; writing – original draft; writing – review and editing. **Kritika Jha:** Conceptualization; visualization. **Dhiraj Poudel:** Visualization. **Saroj Pokharel:** Writing – original draft. **Suraj Aryal:** Resources. **Satish Bajracharya:** Resources. **Bishweshwar Joshi:** Resources. **Asmita Ghimire:** Supervision.

## CONFLICT OF INTEREST STATEMENT

The authors declare no conflicts of interest.

## CONSENT

Written informed consent was taken from patient for publication of the report.

## Data Availability

All the required data are available in the manuscript itself.
